# Ketosis-Prone Type 2 Diabetes: A Case Series

**DOI:** 10.3389/fendo.2019.00684

**Published:** 2019-10-16

**Authors:** Åke Sjöholm

**Affiliations:** Division of Endocrinology and Diabetology, Department of Internal Medicine, Gävle Hospital, Gävle, Sweden

**Keywords:** atypical diabetes, ketosis, Flatbush diabetes, GLP-1–glucagon-like peptide-1, SGLT 2 inhibitor

## Abstract

Ketosis-prone type 2 diabetes (“Flatbush diabetes”) carries features of both classical type 1 and type 2 diabetes and is highly prevalent in African populations. The disease, which is highly ketosis-prone, but neither chronically insulinopenic nor autoimmune, is discussed regarding pathogenesis, diagnosis and treatment from a patient case perspective.

## Introduction

### Diabetes–A Heterogeneous Disorder

Traditionally diabetes is divided into two major groups, type 1 diabetes (T1D) and type 2 diabetes (T2D), along with some more unusual types (see below). All types of diabetes have hyperglycemia as the diagnostic criterion and common denominator, but are otherwise very heterogeneous disorders.

T1D, formerly known as juvenile diabetes or insulin-dependent diabetes, predominantly affects children and young people but can appear at any age. T1D accounts for 5-10% of all diabetes. T1D is an autoimmune disease characterized by the presence of autoantibodies against β-cell antigens (e.g., glutamic acid decarboxylase [GAD-65]), which relatively quickly leads to insulinopenia due to rapid functional suppression and destruction of the pancreatic insulin-producing pancreatic β-cells. The onset is often quick with classic catabolic prodromal symptoms such as polydipsia, polyuria, fatigue, and weight loss. The insulin deficiency confers a strong risk for rapid emergence of severe hyperglycemia with potentially life-threatening ketoacidosis. T1D patients are usually normal weight and not more obese than the normal population. The heredity of T1D is relatively weak.

T2D is a fundamentally different disease than T1D. Unlike the autoimmune etiology of T1D, T2D is much more of a lifestyle disease where lack of exercise and excess caloric intake cause visceral obesity with attendant insulin resistance which in variable degree occurs in 90-95% of T2D patients. In genetically predisposed individuals, the β-cells fail to fully compensate for this increased need with increased insulin production, then overt hyperglycemia and other atherogenic manifestations of insulin resistance (e.g., dyslipidemia, endothelial dysfunction and hypertension) slowly evolve.

LADA (“latent autoimmune diabetes in adults”) can be described as a slow-remitting T1D in somewhat older individuals with progressive autoimmune destruction of the insulin-producing β-cells (1). LADA patients oftentimes have been misclassified as T2D and for several years been treated with oral antidiabetic agents with gradually decreasing effect. To avoid this, one should liberally measure β-cell autoantibodies in normal-weight adult diabetics with slow disease onset or tablet failure.

In addition to these groups are gestational diabetes, secondary diabetes (e.g., after pancreatitis, chronic steroid treatment, etc.) and monogenic diabetes, for example various forms of MODY (“maturity-onset diabetes in the youth”) and neonatal diabetes, which are mainly characterized by β-cell defects (1, 2).

In sub-Saharan Africa, as well as in individuals of African ethnicity (e.g., African-Americans), there is an “atypical” form of diabetes with characteristics of both T1D and T2D (3–10). This type of diabetes, with episodic insulin requirement during spontaneous recurrent ketoses, has been present in Africa for a long time (11–13) and is in the literature under different names: Ketosis-prone T2D (KPT2), T1D-B, “Flatbush diabetes,” type-J diabetes, type 1.5 diabetes, phasic diabetes, and prairie diabetes (4, 7–10).

Written informed consent was obtained from all the below participants for the publication of this report. No potentially identifiable human images or data is presented in this study. The characteristics of the different cases are shown in Table 1.

**Table 1:** Main characteristics of the cases.

	**Case 1**	**Case 2**	**Case 3**	**Case 4**	**Case 5**
Age (years)	45	34	22	36	29
Gender (M/F)	M	F	M	M	M
Country of origin	Congo	Somalia	Sierra Leone	Somalia	Somalia
BMI (kg/m^2^)	29.5	29.8	26.7	22.7	21.1
Insulin dose (U/d)	82	186	34	22	26
Initial HbA_1c_ (mmol/mol)	132	101	153	77	82
Ketosis	++++	+++	+++	++	++
Stimulated C-peptide (nmol/L)	1.44	0.676	“Low”	1.07	1.17
Autoantibodies	Negative x 3	Negative x 2	Negative x 3	Negative x 2	Negative x 2
LDL-cholesterol (mmol/L)	3.7	4.2	5.2	2.9	3.4
Treatment	Sitagliptin	Synjardy Semaglutide	Janumet	Qtern	Sitagliptin
**Follow-up**					
HbA_1c_ (mmol/mol)	57	37	46	40	44
Insulin dose (U/d)	0	18	0	0	0

*M, male; F, female*.

## Case #1

The patient is a man in his forties of Central African ethnicity, previously essentially healthy and on no medications. He was admitted to the hospital because of new onset diabetes with classic catabolic symptoms (polyuria, polydipsia, weight loss) for about 2 months. During this time the patient had lost about 15 kg in weight, from 106 kg to 91 kg (body mass index [BMI] of 34 kg/m^2^ to 29.5 kg/m^2^). There was no known family history of metabolic disease. Upon arrival, the patient was essentially unaffected, and physical examination did not reveal anything remarkable except for abdominal obesity (waist circumference 101 cm). Routine biochemistry, however, showed that he had a pronounced hyperglycemia (non-fasting P-glucose about 40 mmol/l and 3+ glycosuria), and no acidosis but severe ketosis (B-ketones [β-hydroxybutyrate] of 6 mmol/l and 4+ ketonuria [acetoacetate]).

The patient was admitted to a medical ward with standard insulin and fluid replacement therapy. He was at all times and in all respects stable and completely unaffected. A customary four dose insulin regimen was instituted with direct acting insulin analog (insulin aspart) for meals, and long-acting insulin analog (insulin glargine) for bedtime. Glycemic control rapidly normalized, the patient was discharged on 82 U/d of insulin (0.9 U/kg b.w.). During the hospital stay, dyslipidemia was noted with a fS-low-density lipoprotein (LDL)-cholesterol concentration of 3.7 mmol/l, which prompted instigation of atorvastatin (40 mg q.d.). The glycemic long-term control was, as expected, poor with glycated hemoglobin (B-HbA_1c_) of 132 mmol/mol (14.2%, DCCT [diabetes control and complications trial] standard). S-C-peptide concentration was 0.43 nmol/l upon arrival. Blood pressure, renal function, U-albumin/creatinine ratio, and retinopathy screening were normal.

The type of diabetes remained unclear, but the overall assessment still leaned toward T2D. In favor of T2D were the patient's dysmetabolic phenotype of abdominal obesity and dyslipidemia, as well as the absence of acidosis. Against T2D were the relatively rapid onset and catabolic course, but foremost the patient's profound ketosis brings to mind T1D.

After discharge from the hospital results from the analysis of autoantibodies to β-cell antigens (GAD-65, insulinoma antigen 2 [IA-2], zinc transporter 8 [ZnT8]) arrived, all of which were negative. Not least because of the patient's ethnic origin, suspicion of ketosis-prone T2D (KPT2; “Flatbush diabetes”) arose. The past 25 years have seen rising interest in, and awareness of, this atypical form of diabetes and its characteristics (see below).

The patient's insulin requirements decreased significantly and rapidly following his release from the hospital; from 82 U/d at discharge, a week later it had decreased to 14–20 U/d at the visit to the diabetes nurse. The glycemic control was excellent with fP-glucose ranging between 5.5 and 6.5 mmol/l and prandial P-glucose <8.5 mmol/l without hypoglycemia.

Since studies have demonstrated both β- and α-cell dysfunction in KPT2 (14), 2 months after discharge we analyzed fP-glucagon which was found to be 7.9 pmol/l (ref. <18 pmol/l), S-proinsulin 5.2 pmol/l (ref. <16 pmol/l) and S-insulin 3.3 mU/l (23 pmol/l).

We also analyzed glucagon-stimulated C-peptide release such as the proinsulin/insulin ratio, an established surrogate measure of β-cell function. One month after discharge the basal C-peptide level was 0.79 nmol/l, which was significantly better than at the onset of the disease, and 10 min after glucagon (1 mg i.v.) the level had risen to 1.44 nmol/l, a very good response indicating relatively healthy β-cells. Essentially the same C-peptide response remained 5 months after discharge.

Since the human herpes virus type 8 has been proposed to be involved in the pathogenesis of KPT2 (15), and the patient was born in the Congo, we sent serum for polymerase chain reaction (PCR) diagnosis of this but the analysis was negative. Similarly, defects in the enzyme glucose-6-phosphate dehydrogenase (G6PDH), which also causes favism with hemolytic anemia, have been suggested to be involved in the pathogenesis of KPT2 (16). However, the patient's G6PDH activity in serum was normal (4.9 U/g Hb [ref. >4.2 U/g Hb]).

After the discharge, the clinical course has been uneventful and the patient has consistently been well. Upon revisiting 3 months after discharge, the glycemic control had improved considerably with a marked decline in B-HbA_1c_ to 57 mmol/mol (7.4%) despite dramatically reduced insulin requirements, typically <10 U/d (<0.1 U/kg). The patient also had gained 9 kg in weight (BMI 32 kg/m^2^), as expected by the insulin treatment and return to an anabolic metabolism. Furthermore, the lipid profile was significantly improved after the initiation of atorvastatin with fS-LDL-cholesterol of 1.5 mmol/l. He had experienced occasional hypoglycemic symptoms of an adrenergic nature by the extremely small doses (2 U) of meal-time insulin, and was then forced to eat extra carbohydrates, which of course may have contributed to his weight gain. If he took meal-time insulin three times a day, he more often than not suffered hypoglycemia despite the very small doses. Some days he took no insulin at all without problems. He walked 5 to 10 km with his dog almost daily and often experienced adrenergic hypoglycemia which promptly reversed by oral glucose supply. In principle, he never exceeded 8 mmol/l in P-glucose, even after rich meals, at the minimal doses of insulin that he very occasionally took, detailed above. He also stated that postprandial glucose values an hour or two after meals rarely exceeded 7 mmol/l, even without insulin treatment. This indicates a well-preserved β-cell function, probably also including first-phase insulin release as the postprandial glucose peaks were essentially normal.

Because of the frequent and very easily provoked hypoglycemic episodes on minimal doses of insulin, his obesity, and the above clear indications of well-functioning β-cells, it was decided at the revisit to quit the insulin therapy and instead start sitagliptin (100 mg q.d.). By inhibiting the enzyme dipeptidyl peptidase-4 (DPP-4), sitagliptin increases the level of glucagon-like peptide-1 (GLP-1) and we wanted to harness this to therapeutic advantage, as GLP-1 is both a known trophic and anti-apoptotic factor for β-cells [at least in animal models and in human β-cells *ex vivo* (17)] and partly restores the β-cells' “glucose competence” (18, 19), without any risk of hypoglycemia or weight gain. Another reason for the drug choice was that GLP-1 also corrects the overproduction of the diabetogenic and ketogenic hormone glucagon prevailing in KPT2 (20, 21). This regimen has worked very well.

## Case #2

A 34 year old woman from Somalia was referred to our out-patient clinic from primary care because of deranged glycemic control. She was diagnosed with ketotic diabetes in 2009 at the age of 24 and had undergone thyroidectomy in 2016. Her fP-glucose was elevated, oftentimes to 17–19 mmol/l, her B-HbA_1c_ was 101 mmol/mol (11.7%) and fS-LDL-cholesterol concentration 4.2 mmol/l.

Upon physical examination she had a BMI of 29.8 kg/m^2^ but was otherwise unremarkable.

Her medications consisted of insulin detemir 36 U b.i.d. and insulin aspart 38 U t.i.d. (thus a daily insulin dose of 186 U [2.1 U/kg b.w.]), metformin 500 mg t.i.d., repaglinide 1 mg t.i.d. and levothyroxine 125 μg q.d.

She was negative for autoantibodies against GAD-65 and IA-2 and her C-peptide concentration rose from 0.542 to 0.676 nmol/l upon meal stimulation, indicating non-autoimmune non-insulinopenic diabetes.

Metformin and repaglinide were discontinued and replaced by Synjardy (metformin 850 mg and empagliflozin 5 mg) b.i.d. and semaglutide q.w. in escalating dose. Atorvastatin (40 mg q.d.) was also started due to the dyslipidemia. The B-HbA_1c_ target was set to <42 mmol/mol.

Upon revisiting 4 months later, her B-HbA_1c_ was 37 mmol/mol and fS-LDL-cholesterol 2.1 mmol/l. She had lost 7 kg in body weight and reduced her insulin dose to 18 U/d (i.e., a >90% decrease) and so she was referred back to her general practioner (GP) at primary care.

## Case #3

A 22 year old man from Sierra Leone was transferred to our out-patient clinic from a university hospital. He was diagnosed with diabetes in 2016 at the age of 21, presenting with catabolic symtoms (polydipsia, polyuria, weight loss), showed pronounced ketosis (B-ketones [β-hydroxybutyrate] of 4.7 mmol/l) and low C-peptide. The patient was negative for autoantibodies against GAD-65 and IA-2 and the referring university hospital viewed this as a case of autoantibody-negative T1D. His B-HbA_1c_ at diagnosis was 153 mmol/mol (16.8%) and fS-LDL-cholesterol concentration 5.2 mmol/l. For the latter, he was put on simvastatin.

His other medications consisted of insulin glargin 16 U q.d. and insulin lispro 6 U t.i.d. (thus a daily insulin dose of 34 U [0.44 U/kg b.w.]).

Upon his first visit at our hospital, physical examination showed a BMI of 26.7 kg/m^2^ and was otherwise unremarkable. His glycemia had improved substantially by the insulin therapy and he had a B-HbA_1c_ of 66 mmol/mol (8.4%) and fS-LDL-cholesterol concentration 3.5 mmol/l. The patient was negative for autoantibodies against GAD-65, IA-2 and ZnT8 and his C-peptide concentration rose from 0.387 to 0.819 nmol/l upon meal stimulation, indicating a lack of autoimmune diabetes and no insulin deficiency.

All insulin therapy was stopped and replaced with Janumet (metformin 850 mg and sitagliptin 50 mg) b.i.d., and simvastatin was replaced with atorvastatin (40 mg q.d.).

Upon revisiting the diabetes nurse 10 weeks later, the patient's P-glucose never rose above 8 mmol/l, his B-HbA_1c_ was 46 mmol/mol (6.5%) and fS-LDL-cholesterol was 1.5 mmol/l. He was referred to primary care.

## Case #4

A 36 year old man from Somalia was transferred to our out-patient clinic from his GP. He was diagnosed with ketosis-prone diabetes in 2015 at the age of 34. His family history was positive in that his father had T2D-like diabetes. The patient also suffered from bipolar disorder and was a heavy smoker.

His medications consisted of insulin glargin 10 U q.d. and insulin lispro 4 U t.i.d. (thus a daily insulin dose of 22 U [0.29 U/kg b.w.]), olanzapine 15 mg q.d., and slow release valproic acid 500 mg 2 b.i.d.

Upon his first visit at our out-patient clinic, physical examination showed a BMI of 22.7 kg/m^2^ and was otherwise unremarkable. He had a B-HbA_1c_ of 77 mmol/mol (9.5%) and fS-LDL-cholesterol concentration 2.9 mmol/l. He was negative for autoantibodies against GAD-65 and IA-2 and his C-peptide concentration rose from 0.915 to 1.07 nmol/l upon meal stimulation, indicating lack of autoimmunity and a robust insulin production.

All insulin therapy was stopped and replaced with Qtern (saxagliptin 5 mg and dapagliflozin 10 mg) q.d., atorvastatin (40 mg q.d.) and varenicline as anti-smoking therapy.

Upon revisiting the diabetes nurse 4 months later, his B-HbA_1c_ was 40 mmol/mol (5.9%) and fS-LDL-cholesterol was 1.6 mmol/l, but he had not succeeded stopping smoking. The patient was referred back to his GP.

## Case #5

A 29 year old man from Somalia was transferred to our out-patient clinic from the Department of Infectious Diseases after hyperglycemia and ketosis had unexpectedly been found during a negative tuberculosis work up. His family history was positive in that his mother and two uncles had T2D-like diabetes.

His medications consisted of human insulin isophane 16 + 10 U (thus a daily insulin dose of 26 U [0.42 U/kg b.w.]).

Upon his first visit at our out-patient clinic, physical examination showed a BMI of 21.1 kg/m^2^ and was otherwise unremarkable. He had a B-HbA_1c_ of 82 mmol/mol (9.9%) and fS-LDL-cholesterol concentration 3.4 mmol/l. He was negative for autoantibodies against GAD-65 and IA-2 and his C-peptide concentration rose from 0.483 to 1.17 nmol/l upon meal stimulation, indicative of non-autoimmune non-insulinopenic diabetes.

All insulin therapy was discontinued and replaced with sitagliptin (100 mg q.d.) and atorvastatin (40 mg q.d.).

Upon revisiting the diabetes nurse 6 months later, his fP-glucose was usually around 5 mmol/l, prandial P-glucose usually below 7 mmol/l, B-HbA_1c_ 44 mmol/mol (6.3%) and fS-LDL-cholesterol was 1.8 mmol/l. The patient was referred to a primary care health center.

## Discussion

### Epidemiology

Ketosis-prone T2D is common in Africa (3). It also occurs among African-Americans, Latin-Americans, Native Americans, and in parts of Asia (including Japan), but is sparsely reported in Caucasians (22, 23). The name “Flatbush diabetes” arose when a series of cases in 1994 was discovered in the district of East Flatbush in Brooklyn, New York (24). In this paper “ketosis-prone T2D” (KPT2) is consistently used.

In Africa, it is estimated that about 15% of all hospitalized diabetics suffer from KPT2 (3). Africa is incidentally one of the regions where the global diabetes prevalence is expected to increase most; The International Diabetes Federation estimates an increase in manifest diabetes by 100% between 2010 and 2030 in this region and in prediabetes (impaired glucose tolerance) by 75% (3).

KPT2 is estimated to account for nearly 50% of all African-American diabetics diagnosed with ketoacidosis (8). Similarly, ~50% of African-Caribbeans presenting with diabetic ketoacidosis is believed to have KPT2 (9). In the United States, KPT2 is a conspicuously common form of diabetes in regions populated by a high proportion of African Americans (4, 6, 7, 24). Exact prevalence data are lacking, however, as KPT2 relatively recently emerged as a new and diffusely defined entity and due to difficulties to properly classify KPT2 as distinct from T1D and T2D. Nosologically the disorder is since 1997 classified as a separate entity by the American Diabetes Association which calls it idiopathic T1D (25), whereas the World Health Organization calls it T1D-B (26).

### Clinical Picture

KPT2 patients are often middle-aged men (male:female ~3:1) of African ethnicity, positive family history, overweight, and with dysmetabolic traits (abdominal obesity, hypertension, dyslipidemia) [see reviews in (4, 7–10)], a picture thus essentially similar to that of classic T2D. Onset of the disease, however, is often acute and preceded by classic catabolic symptoms. Patients often (but not always) arrive at the emergency department with severe hyperglycemia and severe ketosis, sometimes with acidosis (ketoacidosis), thus a presentation that immediately brings to mind insulinopenia and regular T1D. Confusion tends to arise when the negative outcome of the analyses of autoantibodies against β-cell antigens arrive, oftentimes after the patient has already been discharged and maybe even been given the diagnosis T1D. The initial treatment is also essentially identical to that of T1D, basal-bolus insulin dose titration based on the degree of ketosis and glycemia. The further clinical course, however, mostly resembles T2D.

### Ketosis

Recent elegant mechanistic studies employing metabolomics have made clear that ketosis in KPT2—unlike T1D—is not caused by increased ketogenesis due to the increased flow of free fatty acids to the liver, but rather inhibited oxidation of ketones (27, 28). All this points together but indirectly toward the underlying defects residing in the mitochondrial substrate metabolism. As this is the dominant source of adenosine trisphosphate (ATP) production, which regulates glucose-stimulated insulin secretion in β-cells (29), a defect in this system is also expected to contribute to impaired insulin secretion, which characterizes KPT2. Faulty mitochondrial substrate metabolism could therefore be a pathogenic factor KPT2 shares with mitochondrial monogenic diabetes (30, 31). Molecular genetic studies are underway to more accurately and precisely identify these defects.

In other studies, elevated basal levels of the counter-regulatory pancreatic hormone glucagon were found, indicating also α-cell dysfunction (in addition to β-cell dysfunction) in KPT2 (14), thus a feature KPT2 shares with regular (non-ketotic) T2D (21).

### Genetic Factors

As there is a clear accumulation of KPT2 in certain families, genetic factors are likely important for the onset of the disease. The inheritance is unclear and genetic penetrance unknown. It is not known, therefore, whether KPT2 is polygenic (like regular T2D) or monogenic, but studies are underway to clarify this. Mutations in certain transcription factors of high relevance for β-cell function have been suggested to be linked to KPT2 in some population studies (4, 32), but the significance of these findings remains unclear. Immunogenetically, the mapping of different human leukocyte antigen (HLA) haplotypes revealed an increased prevalence of HLA-DR3 and HLA-DR4 in some KPT2 patients, as well as reduced incidence of HLA class II alleles (DQA^*^03 and DQB1^*^02) (33, 34).

### Pathogenesis: Severe but Reversible β-Cell Dysfunction Caused by Glucotoxicity the Culprit Lesion?

Remarkably often the insulin requirement decreases radically relatively soon after discharge of KPT2 patients. This is probably owing to the β-cell “paralyzed” insulin production in KPT2 (14, 34, 35) quickly recovering when the acute glucotoxicity is corrected by insulin treatment (4, 7–10). The β-cell is very sensitive to glucotoxicity (36), possibly due to low expression of antioxidant enzymes necessary for elimination of pro-oxidative products, notably reactive oxygen species (37). In addition to the parts of the β-cell machinery that senses acute fluctuations in blood glucose levels, and long term expression of the insulin gene deteriorates, through loss of critical transcription factors, by the glucotoxicity (37). This glucotoxicity, which usually entails reversible inhibition of β-cell function (36, 37), seems particularly important in the pathogenesis of KPT2 (38).

The pathogenesis of KPT2 seems, unlike glucotoxicity and also unlike T2D, not to include lipotoxicity (39). On the other hand, it is unclear whether prolonged ketonemia per se exerts negative effects on β-cell function in patients with KPT2. *Ex vivo* studies on human islets of Langerhans from non-diabetic donors, however, suggest this may be the case (40).

Also peripheral insulin resistance has been reported in the acute stage, especially in skeletal muscle, which appears to involve phosphorylation and decreased activation of the enzyme Akt-2 (4). Unlike β-cell function, insulin sensitivity seems not to recover after the acute glucotoxicity is corrected, indicating that it is improved β-cell function that drives the remission process (41).

### Treatment

β-Cell function appears to be fluctuating over time in KPT2, to the extent that up to 75% of patients may be temporarily insulin-independent, sometimes over 10 years (14, 34, 35). In older literature, before the GLP-1 era, sulfonylurea drugs were often chosen to stimulate insulin secretion and thereby prolong normoglycemia in KPT2 (42). Given that many T2D patients over time develop tablet failure on sulfonylurea treatment (43), possibly through apoptotic death in human β-cells (44), and that the natural course of KPT2 (like usual T2D) is characterized by a slowly deteriorating β-cell function (14, 34, 35), more modern therapies were chosen for our patients (see above). There is very little data on which drugs are best in the treatment of KPT2. There is only one (very recent) small randomized clinical trial that shows that sitagliptin and metformin appear equal in terms of maintaining remission in KPT2 (45).

A couple of factors have been shown to predict prolonged remission, insulin independence and preventing relapse of hyperglycemic ketosis: treatment with oral antidiabetic agents after the initial insulin treatment (usually given a few months) instead of only lifestyle measures (diet and exercise), as well as a robust C-peptide response to stimulation (4, 14, 34, 35). The latter may indicate that the outbreak of KPT2 is not due to a loss of β-cells, but rather that their glucose sensing failed, and that the potential for long term remission is related to the β-cell mass.

## Conclusions

The cases presented in this series, although heterogenous, are representative of the spectrum of KPT2. Given that many are young and presenting with ketosis, there is an obvious risk for misclassification as T1D. If KPT2 is misclassified as T1D, these patients risk being unnecessarily and chronically—perhaps for a lifetime—on insulin treatment. Thus, they are subject also to the risks and disadvantages of insulin therapy: hypoglycemia, weight gain, impact on quality of life (requirements for frequent self-monitoring and multiple daily injections), career limitations by exclusion from certain occupations and possible ramifications for insurance coverage.

Use of SGLT2 inhibitors is associated with an increased risk of ketoacidosis, oftentimes at or near normoglycemia (46). Caution should be exerted using SGLT2 inhibitors in KPT2 patients, given their risk of recurrent ketosis and temporary insulinopenia. Patients should be given oral and written information on the signs and symptoms of ketoacidosis, preventive measures and risk situations and actions to take in case of ketosis *et cetera*. They should be prescribed devices and test strips for monitoring of ketonemia. Excellent papers have recently been published on this topic (47, 48).

## Author Contributions

ÅS cared for the patients, wrote, and edited the manuscript.

### Conflict of Interest

The author declares that the research was conducted in the absence of any commercial or financial relationships that could be construed as a potential conflict of interest.
